# Comprehensive chromosomal aberrations in a case of a patient with *TCF3-HLF*-positive BCP-ALL

**DOI:** 10.1186/s12920-020-0709-y

**Published:** 2020-04-03

**Authors:** Monika Lejman, Monika Włodarczyk, Joanna Zawitkowska, Jerzy R. Kowalczyk

**Affiliations:** 1 Laboratory of Genetic Diagnostics, Department of Pediatric Hematology, Oncology, and Transplantology, Medical University of Lublin, ul. Antoniego Gębali 6, Lublin, Poland; 2 Laboratory of Genetic Diagnostics, Medical University of Lublin, Lublin, Poland; 3 Department of Pediatric Hematology, Oncology, and Transplantology, Medical University of Lublin, Lublin, Poland

**Keywords:** Acute lymphoblastic leukaemia, Case report, *TCF3-HLF*, Molecular abnormalities, Gene fusion, *RB1*

## Abstract

**Background:**

The use of high-throughput analytical techniques has enabled the description of acute lymphoblastic leukaemia (ALL) subtypes. The *TCF3-HLF* translocation is a very rare rearrangement in ALL that is associated with an extremely poor prognosis. The *TCF3-HLF* fusion gene in the described case resulted in the fusion of the homeobox-related gene of *TCF3* to the leucine zipper domain of *HLF*. The *TCF3-HLF* fusion gene product acts as a transcriptional factor leading to the dedifferentiation of mature B lymphocytes into an immature state (lymphoid stem cells). This process initiates the formation of pre-leukaemic cells. Due to the rarity of this chromosomal aberration, only a few cases have been described in the literature. The advantage of this work is the presentation of an interesting case of clonal evolution of cancer cells and the cumulative implications (diagnostic and prognostic) of the patient’s genetic alterations.

**Case presentation:**

This work presents a patient with diagnosed with *TCF3-HLF*-positive ALL. Moreover, the additional genetic alterations, which play a key role in the pathogenesis of ALL, were detected in this patient: deletion of a fragment from the long arm of chromosome 13 (13q12.2-q21.1) containing the *RB1* gene, intragenic deletions within the *PAX5* gene and *NOTCH1* intragenic duplication.

**Conclusions:**

A patient with coexistence of chromosomal alterations and the *TCF3-HLF* fusion has not yet been described. Identifying all these chromosomal aberrations at the time of diagnosis could be sufficient to determine the cumulative effects of the described deletions on the activity of other oncogenes or tumour suppressors, as well as on the clinical course of the disease. On the other hand, complex changes in the patient’s karyotype and clonal evolution of cancer cells call into question the effectiveness of experimental therapy.

## Background

Among childhood leukaemias, B-cell precursor acute lymphoblastic leukaemia (BCP-ALL) has the highest rate of incidence, accounting for approximately 80% of childhood leukaemias in Western countries [1]. Chromosomal aberrations in ALL can lead to gene fusion, resulting in the expression of chimeric fusion proteins. The t(17;19)(q22;p13) translocation leading to the fusion gene *TCF3-HLF* is a very rare aberration that is probably related to an unfavourable prognosis and is associated with relapse and death within 2 years from diagnosis [2, 3].

## Case presentation

**Fig. 1:**
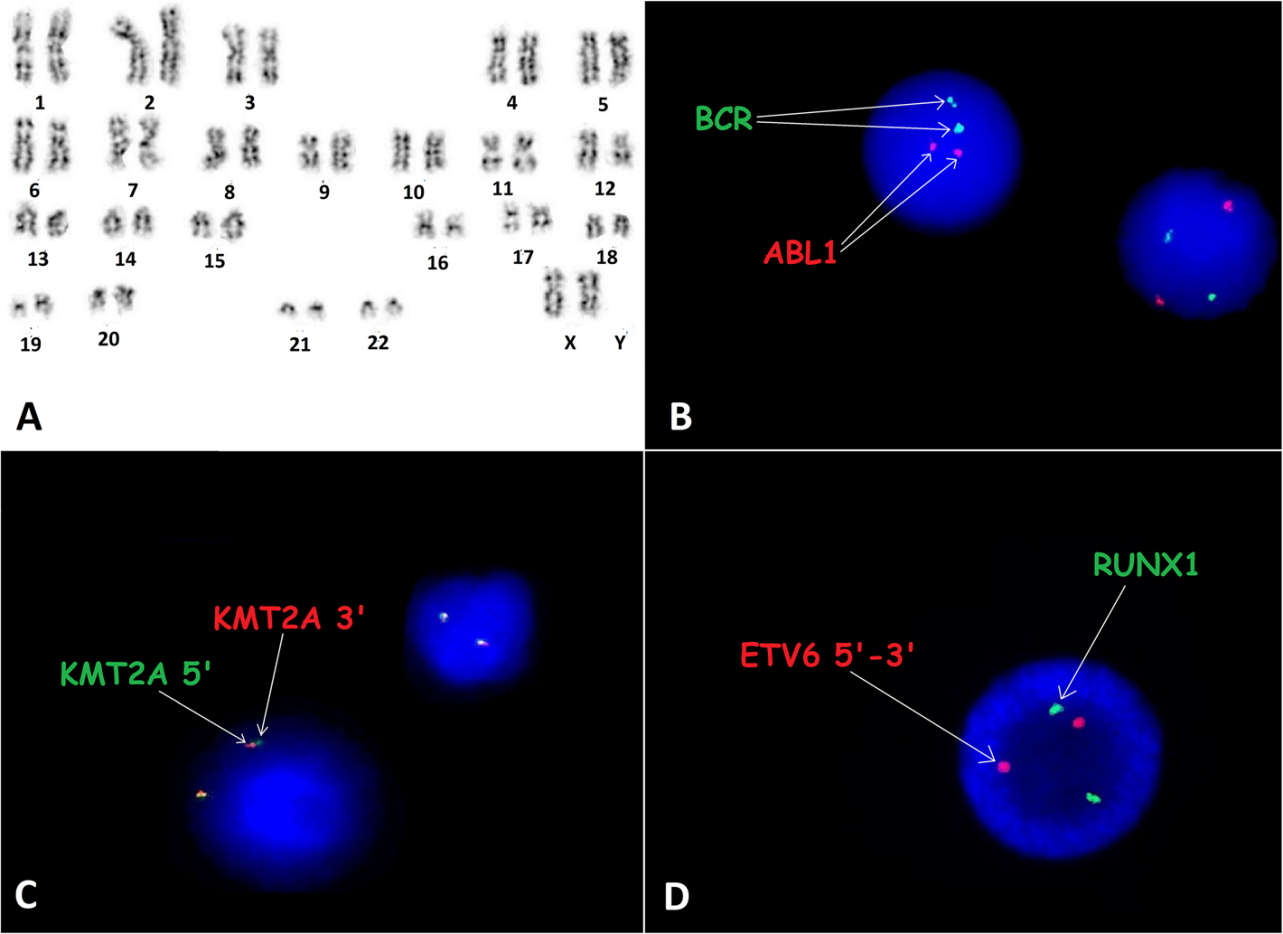
Cytogenetic features at diagnosis. **a** GTG band staining study of the patient revealed 46,XX. **b**, **c**, **d** Results of FISH tests with *BCR/ABL1*, *KMT2A* and *ETV6/RUNX1* probes. FISH was performed on interphase nuclei using probes (Cytocell Ltd., Oxford Gene Technology, Cambridge, United Kingdom) according to the manufacturer’s recommendations. Images were captured by an Olympus BX41TF microscope equipped with a Jenoptik camera and analysed with Isis Software (MetaSystems)

A 15-year-old girl was admitted to the Department of Paediatric Haematology, Oncology and Transplantology, Medical University of Lublin, Poland, because of petechiae and bruising on the lower extremities and a pale complexion. There were no comorbidities, such as obesity, diabetes, or bronchial asthma as well as no significant findings in the patient’s family history. The child was diagnosed with common B-cell precursor ALL, and chemotherapy was started in August 2015, according to the ALL IC-BFM 2009 protocol (ALL Intercontinental-BFM 2009). She was classified as being in the intermediate risk group (age > 6 years, white blood cells < 20,000/μl; a good response to steroids: on day 8 blast count in peripheral blood < 1000/μl; myelogram on day 15: 5% blasts; and minimal residual disease (MRD): > 0.1 < 10%). No central nervous system involvement was observed. According to the ALL IC-BFM 2009 protocol, GTG band staining and fluorescenc*e* in situ hybridization (FISH) tests were performed with the use of molecular probes *BCR/ABL1*, *KMT2A* and *ETV6/RUNX1* (Vysis, Abbot Molecular, Illinois, USA) at the time of diagnosis. The arrangement of signals from the probes used indicated the lack of chromosomal aberrations (Fig. 1). The induction phase of therapy was complicated by post-steroid diabetes and intestinal perforation (a stoma was necessary). This caused a month-long break in chemotherapy. Before continuation of the therapy, the myelogram presented 2% blasts. The consolidation and reinduction phases of therapy were tolerated well enough. Intensive chemotherapy was completed in June 2016. Maintenance treatment was started in July 2016. The surgery to close the stoma was performed at the same time.

**Fig. 2:**
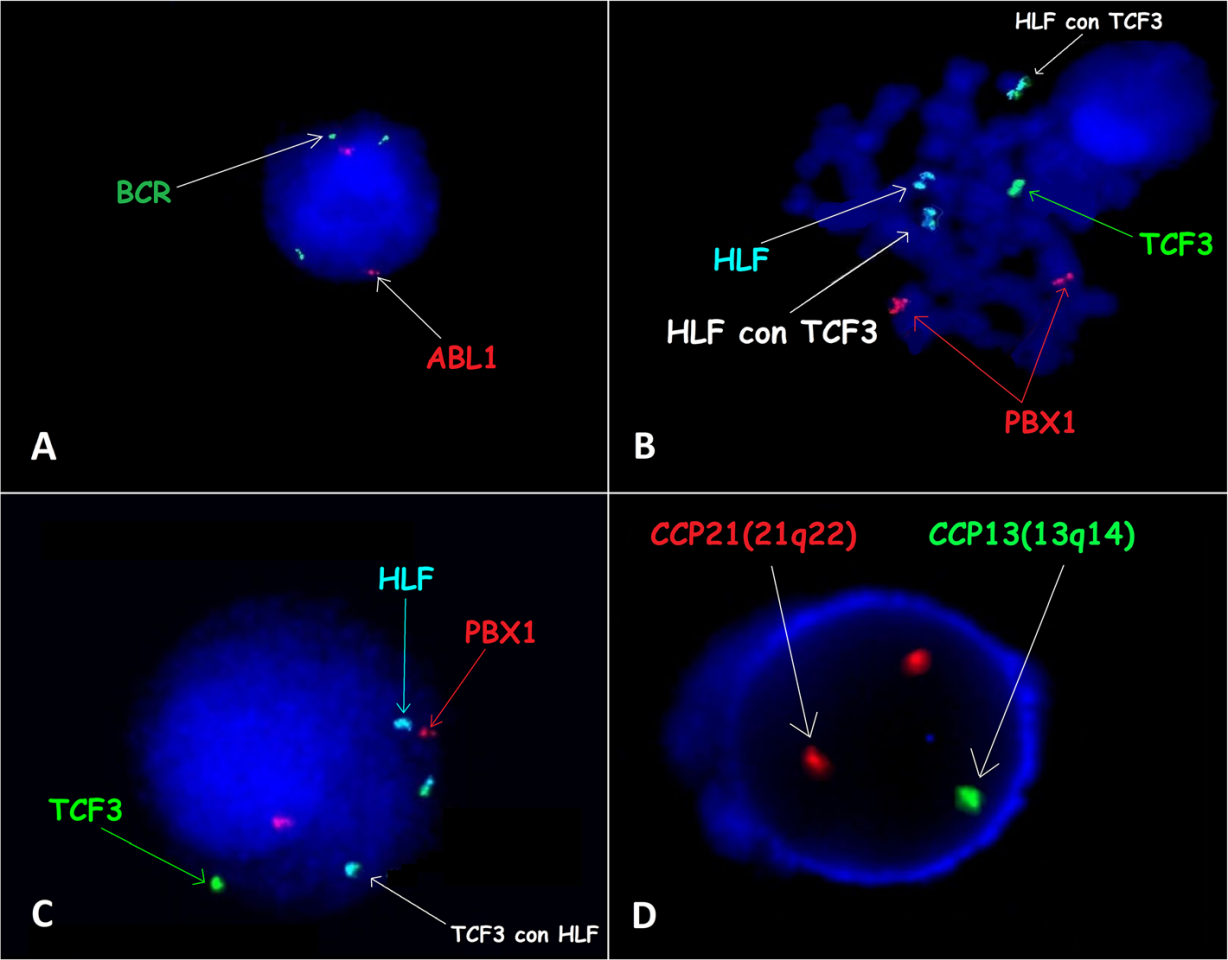
Cytogenetic features at diagnosis and relapse. **a** Image of the FISH results with the BCR/ABL1 probe revealing an additional green signal from the *BCR* locus on chromosome 22. Images of metaphases FISH with the TCF3/PBX/HLF probe in samples from diagnosis (**b**) and relapse (**c**). Image of interphase FISH with CCP13/CCP21 probe in samples from diagnosis (**d**) revealing a lack of 13q deletion genes: *MED4, ITM2B, RB1, RCBTB2,* and *CYSLTR2*). FISH was performed on cells in metaphase using probes (Cytocell Ltd., Oxford Gene Technology, Cambridge, United Kingdom) according to the manufacturer’s recommendations. Images were captured by an Olympus BX41TF microscope equipped with a Jenoptik camera and analysed with Isis Software (MetaSystems)

The patient was readmitted to the clinic in August 2016 due to numerous petechiae, bruising and thrombocytopenia. The bone marrow aspirate smear revealed very early relapse of ALL. Treatment was initiated, according to the IntReALL 2010 HR protocol. Genetic tests were performed again using classical cytogenetics and FISH, and the presence of chromosomal aberrations was again not found, with the exception of an additional signal from chromosome 22 (Fig. 2a). The HIA treatment course applied in weeks 1–4 included dexamethasone, vincristine, methotrexate, PEG-asparaginase, cytarabine, and intrathecal methotrexate. The HIA treatment was complicated by pancreatitis and bradycardia. The myelogram presented 75% blasts on day 28. The patient received the HIB treatment course (including dexamethasone, clofarabine, cyclophosphamide, etoposide, intrathecal methotrexate, cytarabine, and prednisolone), but the treatment was unsuccessful. The patient died due to disease progression. Additional analyses of the patient’s genetic material are a routine procedure used in patients with the short survival in whom a very aggressive disease course and resistance to treatment were observed. Following the data discussing cases of the patients with a similar course of the disease, we used probes for the *TCF3-HLF*. Retrospective genetic examinations included additional genetic testing (FISH and microarray) of material collected at the time of diagnosis, which showed the presence of the *TCF3-HLF* (Cytocell, Cytocell Ltd., Oxford Gene Technology, Cambridge, United Kingdom) fusion in 89% of blasts (Fig. 2b and c). Additionally, tests were performed using a CytoScan HD microarray (Applied Biosystems, part of Thermo Fisher Scientific, Waltham, MA) for copy number variation (CNV) analysis, which showed additional changes in the form of a deletion of a fragment of the long arm of chromosome 13 (13q12.2-q21.1) containing the *RB1* gene, intragenic deletions within the *PAX5* (exons 1–6) gene and *NOTCH1* intragenic duplication (exons 3–34). The 13q, *PAX5*, and *NOTCH1* alterations and the *TCF3-HLF* fusion were present in the same cell clones. The absence of aberrations of 13q in the karyotype analysis indicates that only cells with a normal karyotype have grown in the cell culture. Deletion of 13q was confirmed in interphase cells by FISH (CCP13/CCP21 FISH Probe Kit, CytoTest). Blasts with 13q deletion accounted for 86.4% of the myelogram at initial treatment. Despite molecular studies that revealed a partial 13q deletion, this aberration was not found in the karyotype analysis (Fig. 1a). Unfortunately, the number of protected samples from the patient was not enough for further tests, including RT-PCR.

**Fig. 3:**
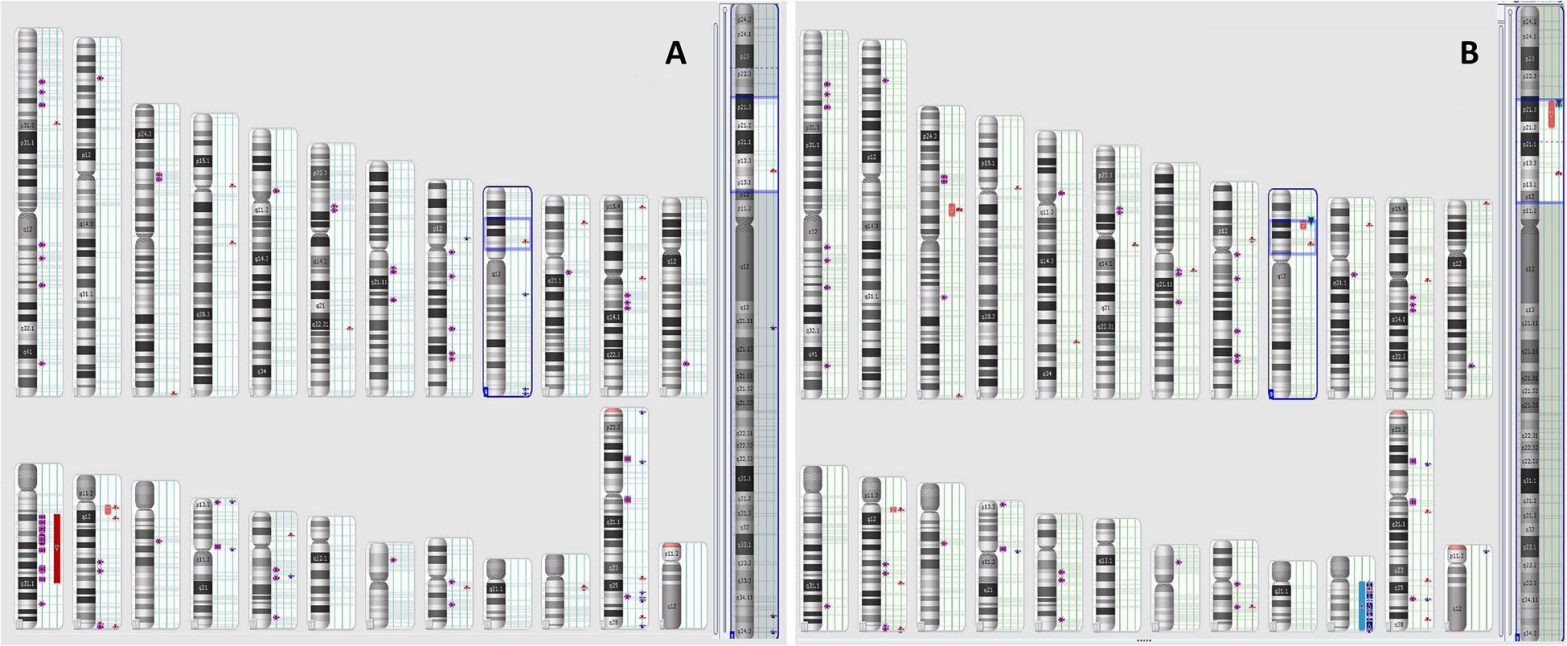
Karyoviews from microarray results at the time of diagnosis and relapse. **a** Microarray results revealing a deletion of fragment of the long arm of chromosome 13 (13q12.2-q21.1) containing the *RB1* gene (red box), *PAX5* intragenic deletion and *NOTCH1* intragenic duplication (both on chromosome 9). **b** Microarray results revealing that 13q deletion was not found in samples from relapse. In addition to the *PAX5* and *NOTCH1* alterations, *CDKN2A/B* deletion (red box) and 22 trisomy (blue box) were also observed. Asterisks correspond to deletion (red colour), duplication (blue colour) and loss of heterozygosity (purple colour)

Additionally, all the tests mentioned above (GTG band staining, FISH, and microarray) were performed on samples from relapse. The results obtained in samples from relapse suggest the clonal evolution of cancer cells because no 13q deletion was found, while the myelogram presented 77% blasts with the *TCF3-HLF* fusion (Fig. 2c and d). Moreover, the microarray test revealed deletion of the *CDKN2A* and *CDKN2B* genes (Fig. 3a and b).

The detected translocation t(17;19) is included in the new treatment protocol (AIEOP BFM ALL 2017) as qualifying the patient for the high risk group and for experimental therapy.

## Discussion and conclusions

The described case concerns the translocation t(17;19)(q22;p13), which occurs in < 1% of BCP-ALL cases [4, 5]. The presence of this genetic change is associated with very poor prognosis (five-year survival without relapse is 0%); therefore, it is an independent negative prognostic factor. The translocation t(17;19) is associated with poor response to treatment and early relapse of the disease, even after the patient receives haematopoietic stem cell transplantation [4, 6]. Symptoms associated with t(17;19) include coagulopathy and hypercalcemia [5].

*TCF3* is a transcription factor responsible for the maturation of B lymphocytes; therefore, translocation involving this gene interferes with transcriptional activity throughout the signalling pathways associated with this process [7, 8]. The *HLF* gene encodes a bZIP (basic leucine zipper) transcription factor, a member of the proline and acid-rich protein family [7, 9].

**Fig. 4:**
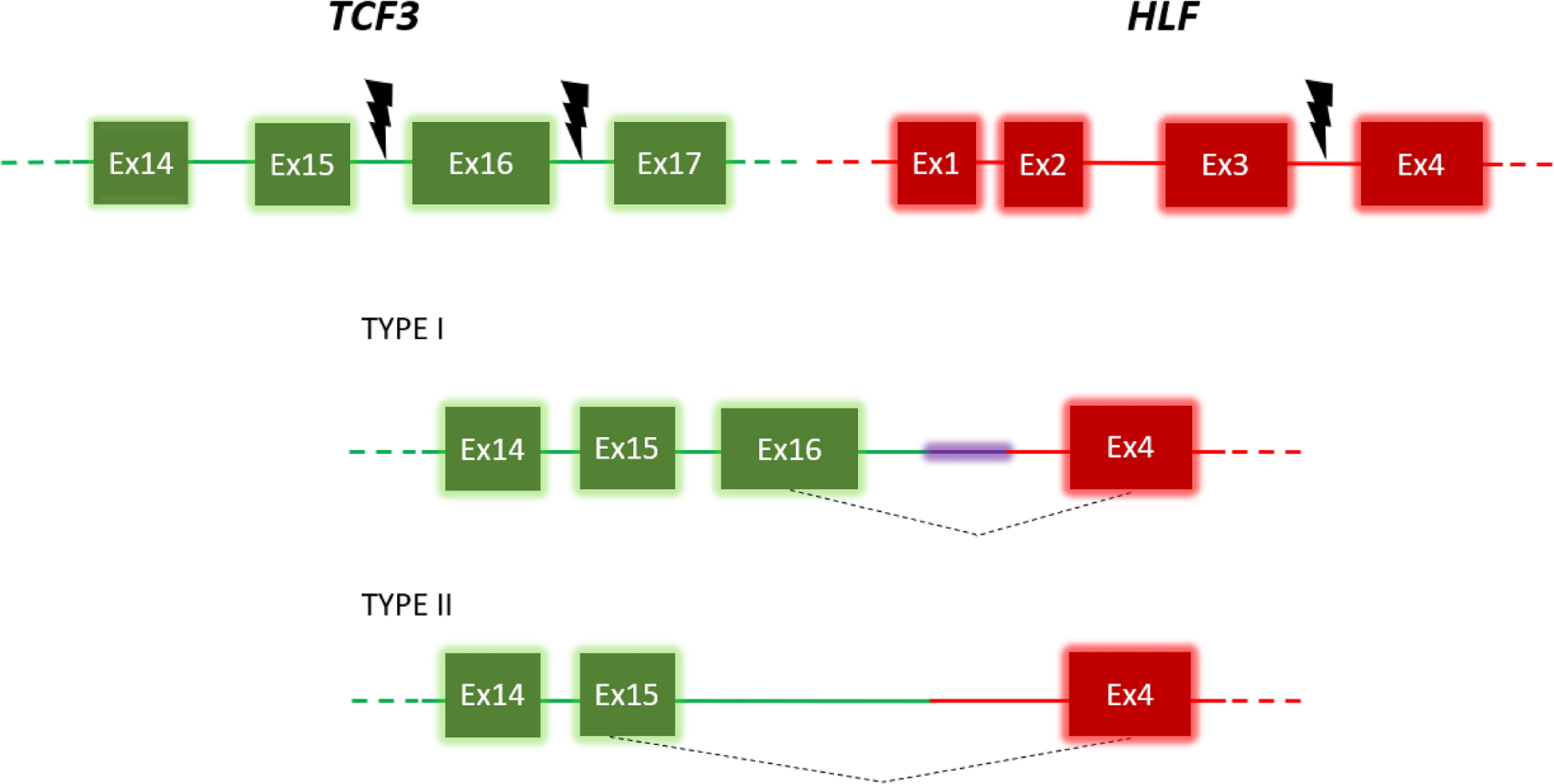
The schemes around the breakpoints of two different types of *TCF3-HLF* fusion transcripts are depicted. Two breakpoints clustering in two *TCF3* intronic regions are distinguished. At the transcript level, type I translocation results in joining *TCF3* exon 16 to *HLF* exon 4. Moreover, intronic sequences, new splice sites and inserted non-template sequences are attached to the *HLF* gene (purple shadowed line). Implications resulting from the insertion of certain sequences have not yet been studied. Type II translocation occurs downstream of *TCF3* exon 15. In this case, *TCF3* exon 16 is not part of the fusion transcript. Boxes correspond to exonic regions of the *TCF3* gene (green) and the *HLF* gene (red). Lines correspond to intronic regions of the *TCF3* gene (green) and the *HLF* gene (red). Additional upstream and downstream exons of the *TCF3* and *HLF* gene are not graphically represented (broken coloured lines). Lightning bolts represent intronic breakpoints

In our case, the *TCF3-HLF* fusion was a result of the translocation of the homeobox related gene *TCF3* to the leucine zipper domain of *HLF*. *TCF3-HLF* acts as a transcription factor leading to the dedifferentiation of mature lymphocyte B into an immature state (lymphoid stem cells) (Fig. 4). This process initiates the formation of pre-leukaemic cells [3, 10]. However, another translocation involving *TCF3* is t(1;19)(q23,p13). This *TCF3-PBX1* fusion accounts for approximately 5–10% of childhood ALL cases [11]. In this case, *PBX1* acts as a transcription factor through fusion of the transcriptional activator domain of *TCF3* with *PBX1* on 1p23. In contrast to *TCF3-HLF* ALL, the 5-year survival rate of *TCF3-PBX1* ALL is 80–90% with intensive chemotherapy [11, 12]. Differences between both ALL subtypes may be related to the DNA methylation process, which is responsible for the regulation of transcription factors and the structure of chromatin. Over 7000 CpG sites were differentially methylated between *TCF3-HLF* ALL and *TCF3-PBX1* ALL, and these sites correlated with other gene expression levels (including genes involved in leukaemogenesis) [13].

In cases with established *TCF3-HLF* fusion, mutually exclusive deletions of the *PAX5*, *VPREB1* and *BTG1* genes are also observed [3]. Genetic disorders associated with t(17;19), which also include mutations within the *RAS* pathway genes (*NRAS*, *KRAS*, and *PTPN11*) may contribute to poor response to treatment [3]. In our case, no changes in any of these genes were observed. However, we found intragenic deletions of *PAX5.* Expression of the *TCF3-HLF* fusion gene alone is not sufficient for neoplastic transformation, as implied by studies of *TCF3-HLF* transgenic mice, which do not develop the human phenotype [3, 14].

Despite the rarity of childhood ALL, t(17;19) is being increasingly recognized as a clinically significant chromosomal rearrangement. According to drug screening results, *TCF3-HLF-*bearing tumour cells obtained from paediatric patients show resistance to standard therapy, including nucleotide analogues (e.g., cytarabine) and mitotic spindle inhibitors (e.g., vincristine) but are sensitive to glucocorticoids [15]. Studies to date show that the use of tyrosine kinase inhibitors or CD19-directed immunotherapy can improve the quality of remission in *TCF3-HLF*-positive ALL patients [16]. Moreover, *TCF3-HLF*-positive cells are sensitive to PARP inhibitors (olaparib and veliparib) in vitro*,* but monotherapy with these drugs was not effective in in vivo studies [17].

Moreover, in this case the presence of additional genetic alterations, which play a key role in the pathogenesis of ALL, was detected. Common submicroscopic abnormalities associated with BCP-ALL include alterations within *PAX5* – a transcription factor that regulates the normal development and maturation of B-cells. Regardless of which fragment of the *PAX5* is deleted, this process results in protein dysfunction [18].

Loss of 13q is associated with a higher risk of relapse. In this case, due to deletion of the long arm of chromosome 13, the negative cell cycle regulator *RB1* was deleted. Loss of function of *RB1* due to gene deletion is found in approximately 6% of BCP-ALL patients. Deletions within *RB1* are found in approximately 50% of all patients who also have 13q loss. The molecular consequences of this recurring deletion are unclear; however, the loss of *RB1* is associated with a disruption of the function of other tumour suppressors [19]. In our case, the *RB1* deletion was limited to blasts. The karyotype analysis of the patient’s parents did not find aberrations of chromosome 13. Deletion of 13q may confer an increased risk of treatment failure. However, to date the deletion of 13q has not been shown to be an independent prognostic indicator [19, 20]. This case describes loss of *RB1* in a patient with *TCF3-HLF* fusion for the first time.

Abnormalities within the *NOTCH1* gene are widely described in T-cell acute lymphoblastic leukaemia (T-ALL). Alterations within *NOTCH1* are characteristic of approximately 50–70% of T-ALL patients, and most of these abnormalities are *NOTCH1* activating mutations. These mutations often result in enhanced signal transduction of the NOTCH1 pathway due to stabilization of the active form of NOTCH1 and impaired degradation of this molecule. Activating mutations of *NOTCH1* confer an increased ability to self-renew to cancer cells. Intragenic *NOTCH1* deletions account for approximately 2.5% of all alterations identified in this gene in T-ALL patients [21]. However, there is a lack of reports about the frequency of deletions in *NOTCH* genes and their effects on the pathogenesis of BCP-ALL. The high percentage of *NOTCH1* activating mutations in patients with T-ALL prompted researchers to conduct clinical studies on the effectiveness of the *NOTCH1* signalling blockers γ-secretase inhibitors (GSIs). According to the results obtained so far, the combination of a GSI and glucocorticoids in T-ALL therapy may have increased efficacy and decreased toxicity [22]. Moreover, the use of a GSI together with conventional chemotherapy resulted in potentiated drug-induced cell death in B-ALL cells by upregulating intracellular levels of reactive oxygen species [23].

The use of microarrays also allowed for the identification of other unbalanced submicroscopic abnormalities. *CDKN2A/B* deletions are also commonly identified in patients with ALL. The *CDKN2A/B* genes act as tumour suppressors, and monoallelic or biallelic deletions of these genes are often associated with poor survival. Furthermore, the loss of *CDKN2A/B* is often associated with a higher risk of relapse. Deletions of *CDKN2A/B* are often accompanied by a higher WBC count and older patient age at the time of diagnosis. The *CDKN2A/B* and *PAX5* deletions are also associated with high-risk cytogenetics, e.g., *BCR-ABL1* translocation. Moreover, as a result of the constitutive activation of *NOTCH* associated with neoplastic transformation, *CDKN2A* is deleted. Additionally, alterations within *PAX5* often correlate with the occurrence of deletions in *CDKN2A/B* [19, 22].

Recurrent complex changes in the patient’s genotype, including *CDKN2A/B* deletion and *NOTCH1* duplication together with maintained *PAX5* deletion, may have had an additional impact on the poor outcome and rapid death from disease progression. Furthermore, the clonal evolution of cancer cells with the *TCF3-HLF* fusion and the genetic alterations described above indicates that experimental treatment might have been ineffective.

A comprehensive analysis of genetic abnormalities may indicate new therapeutic targets, which is of great importance for patients who have not responded appropriately to today’s treatment regimens. Due to the rarity of t(17;19), it is difficult to assess the true value of using immunotherapy to improve the outcome of this ALL subtype. *TC3F-HLF*-positive ALL remains an incurable disease. The most effective therapy in the first line of treatment of high-risk patients remains intensified chemotherapy and haematopoietic stem cell transplantation. Identifying all these chromosomal aberrations at the time of diagnosis could be sufficient to determine the effect of the described deletions on the activity of other oncogenes or tumour suppressors, as well as on the clinical course of the disease. In summary, the described case emphasizes the need to diagnose haematological cancers using high-throughput analytical techniques to obtain a comprehensive picture of genetic alterations.

## Data Availability

The datasets generated and/or analysed during the current study are available in the Gene Expression Omnibus (GEO) repository, https://www.ncbi.nlm.nih.gov/geo/query/acc.cgi?acc=GSE147280.

## Abbreviations

ALL: Acute lymphoblastic leukaemia
HSCT: Haematopoietic stem cell transplantation
BCP-ALL: B-cell precursor acute lymphoblastic leukaemia
MRD: Minimal residual disease
FISH: Fluorescenc*e* in situ hybridization
CNV: Copy number variation
T-ALL: T-cell acute lymphoblastic leukaemia
HIA treatment course: Dexamethasone, vincristine, methotrexate, PEG-asparaginase, cytarabine, intrathecal methotrexate
HIB treatment course: Dexamethasone, clofarabine, cyclophosphamide, etoposide, intrathecal methotrexate, cytarabine, prednisolone
